# Is Earlier Better? The Relationship between Age When Starting Early Intervention and Outcomes for Children with Autism Spectrum Disorder: A Selective Review

**DOI:** 10.1155/2020/7605876

**Published:** 2020-08-03

**Authors:** Patricia O. Towle, Patricia A. Patrick, Tamique Ridgard, Sofia Pham, Jaime Marrus

**Affiliations:** ^1^Westchester Institute for Human Development, New York Medical College, Valhalla, New York, NY, USA; ^2^Department of Health Science and Practice, New York Medical College, Valhalla, New York, NY, USA

## Abstract

Although the conventional wisdom is that “earlier is better” when it comes to intervention for children with ASD, it is not clear what evidence exists to support this notion. This review examined a group of studies that addressed outcomes for young children with ASD who started early intervention at a range of ages. The review was selective by including only papers that examined the age of initiation of treatment as well as baseline cognitive, language, or adaptive level and, in addition, employed a method to control for the covariance between early ability level and age of beginning intervention. Fourteen studies were identified and then compared on methods and outcomes. The support for “earlier is better” was mixed, but it was clear that complex relationships among predictor variables need to be explored in order to understand the role of age of starting early intervention for later outcomes.

## 1. Introduction

Autism spectrum disorder (ASD) is a neurobehavioral disorder that significantly affects social interaction, communication, and interests. The Centers for Disease Control and Prevention recently reported an estimate that 16.8 per 1,000, or one in 59, children aged 8 years have ASD [1]. One of the most important recent research and clinical advances in the field has been the ability to diagnose ASD at increasingly younger ages; the current consensus is that most children with ASD can be diagnosed by 24 months [2–4]. Early diagnosis is now strongly encouraged due to the mounting evidence for the positive effects of early intervention [5–7], which is seen as one of the keys to mitigating the lifelong effects of ASD and associated costs [8, 9]. A corollary consensus is that the earlier a child starts in an intervention program, the better the outcome. However, it is not clear what direct evidence exists demonstrating this to be the case [10, 11].

There are several strong and interrelated rationales for the assertion that earlier is better when it comes to intervention. The first relates to early *neuroplasticity*, with the birth-to-three period considered a peak neuroplastic phase due to the rate of synaptic formation [12]. The human brain is not functionally mature at birth, but requires extensive interaction with the environment (i.e., *experience*) for elaborated synaptic connections and cortical specialization in combination with genetically programmed neuromaturation [13, 14]. Early intervention can be seen as highly specialized experience that may shape and even correct patterns being formed during the birth-to-three period.

The concept of early neuroplasticity is further elaborated by the notion of *critical* or *sensitive periods*—times during which the brain is primed for specific areas of learning via experience combined with organismic development; an associated feature is that the window incrementally closes up to a certain point. There is now a substantial body of evidence, generated through the Baby Siblings Research Consortium, showing that ASD features in infancy are emerging at the same time that typical early social and communication skill sets are building and consolidating [15–17]. The earliest observable ASD symptoms usually become evident from 12 to 18 months of age. Yet it is highly likely that the basic developmental processes have derailed even earlier—at a prodromal stage—and these processes are both resulting in observable symptoms and interfering with further typical development [18].

There are now several models of early autism etiology such as the “social motivation” hypothesis [19–21], as well as those based on broader disturbances in attention, biologic motion, and sensory perception. Each of these has accumulating imaging and psychophysical evidence of associated neurologic differences between controls and infants who are later diagnosed with ASD [22, 23]. Yet each model incorporates the concept of a *developmental cascade*, wherein disturbances of one phase of development significantly interfere with the successful acquisition of those subsequent, mirrored by early neurologic differences that interfere with further cortical specialization and typical skill development [24, 25].

Considering these lines of evidence and theory, a strong argument can be made for intervention for children with ASD to take place as close to early critical periods as possible and certainly when the first autism symptoms appear. It follows that the more time that goes by, the harder it will be to remediate neural circuitry and behavioral expression for more optimal behavioral patterns. It remains unclear, however, to what extent the specific question of the relative effects of earlier intervention has been asked and answered definitively [11, 26, 27].

This paper examines the extant literature on the role that age of starting early intervention plays in the varying outcomes for children with ASD. Studies were identified that included age of starting intervention as a predictor of later capability levels of children diagnosed early with ASD. However, two lines of research informed a selective rather than a broader review. The first is that there is clear evidence that one of the strongest early predictors of outcome in ASD is baseline cognitive functioning [28, 29]. Other significant predictors have likewise reflected early skill levels, such as early language [30], baseline play [31], and social interaction capabilities [32] as key determinants in the long-term outcomes for children with ASD. Therefore, to be considered in this review, along with age of starting early intervention, the study needed to (1) also include as a predictor at least baseline cognitive or language level and (2) determine other predictors' covariance with age of starting intervention, and in addition, employ a statistical or design method for controlling for shared variance between age of entry into EI and baseline ability level in relation to outcome variables.

These criteria were considered essential because research has shown that there are important child features that increase the likelihood of earlier diagnosis (and thus start of intervention), such as presence of obvious repetitive behaviors [33, 34], greater delays associated with eventual intellectual disability [33], and more severe communication and social delays [35]. As mentioned, these are features that will have a strong influence on later outcomes and may override the individual effect of starting age. Several environmental variables also have been shown to influence age of diagnosis, including parental education level [35], geographic region reflecting the density of professional resources, parental attribution of delays, degree of previous interactions with health systems [36], and cohort effects [33, 35].

These influences make it possible that the younger vs. older children (when beginning EI) in a study may not be similar in terms of strongly influential features such as baseline cognitive level or well-developed parental advocacy. The methods to address these possible confounds in the papers reviewed were anticipated to be a multiple regression design or other strategies that controlled for shared variance among predictors (e.g., ANCOVA).

After a systematic search, the included studies were characterized in terms of research design, participant features, follow-up period, predictor variables, and data analytic methods. Results across studies were synthesized and outcomes interpreted with a focus on the role played by age of entering early intervention.

## 2. Method: Literature Search and Criteria for Inclusion

The electronic databases of PubMed, PsychINFO, Education Resources Information Center (ERIC), and Google Scholar were searched from 1995 to 2018, using search terms that included various combinations of “infants,” “young children,” “at-risk,” “autism,” “autism spectrum disorder,” “age,” “early intervention,” “prediction,” “longitudinal,” and “outcomes.” Gray literature was not searched. Hand searches also were completed of reference lists from included articles as well as those from four reviews and three meta-analyses. Criteria for inclusion were that the study was published in a peer-reviewed journal, was available in English, used either DSM-IV, DSM-IV-TR, or DSM-5 criteria for autism or ASD diagnosis, and included age of starting early intervention as one focus of inquiry along with other key predictors. As explained in the Introduction, studies for detailed review also needed to inquire past simple group comparisons or zero-order correlations from predictors to outcome, include several other predictor variables besides initial age, and use statistical techniques for controlling for their shared variance.

## 3. Results

### 3.1. Study Selection

**Figure 1:**
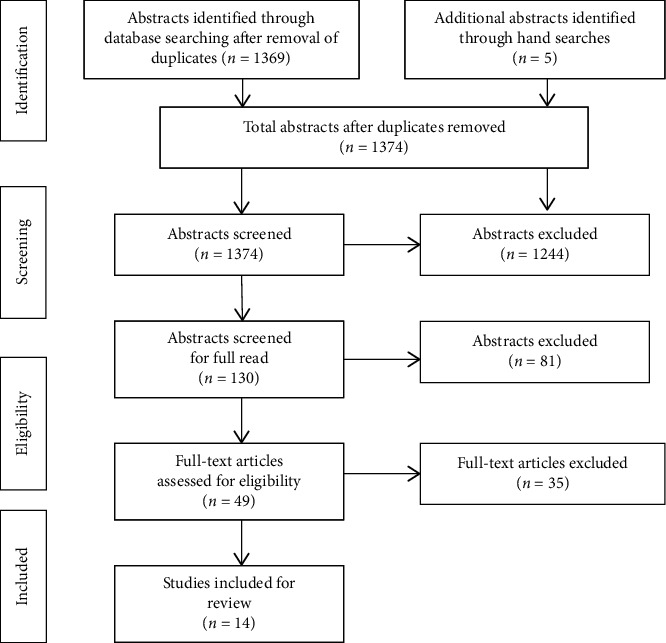
Flow diagram of article screening and review.

The PRISMA flow diagram for the process of selecting final studies for inclusion is shown in Figure 1. After an initial screen to determine that only children with ASD were involved, a treatment component was applied and outcome of treatment was the focus, and age of entry into intervention was included, 1,244 were excluded. Two raters (authors PT and PP) then independently judged whether the abstract should be chosen for a full read. These raters achieved moderate agreement at this stage of study selection (*K* = 0.64; 95% CI: 0.49, 0.79). Disagreements were resolved through discussion, and 130 articles were read thoroughly and independently by the same two raters; strong agreement was achieved at this final stage (*K* = 0.89; 95% CI: 0.74, 1.0), with the result of 14 articles to be included for the review.

### 3.2. Systematic Review of Studies

#### 3.2.1. Range of Studies and Features

Of the 14 included studies, five were from the United States, two were from Canada, four from Australia, and one each from Israel, Spain, and Norway. Five studies looked exclusively at infants and toddlers, while five spanned the birth-to-three and preschool age (3–5 years), and four studies included preschoolers as well as children up to 7 years of age.

Table 1 shows the following for the 14 studies: research design, sample size, age of participants, length of the follow-up period, and measurements used. There were 12 intervention studies and two longitudinal studies. Sample sizes ranged from 24 to 332. The follow-up period after intervention ranged from 12 weeks to 17 years.

In addition to child age at initiation of treatment, all of the studies assessed cognitive functioning, 12 assessed adaptive behavior skills, 13 measured autism severity, nine assessed language skills, and seven measured some dimension of early social functioning. For cognitive measurement, a common instrument was the Mullen Scales of Early Learning (MSEL) [37]. Other measurements used were the following norm-referenced instruments: the Differential Ability Scales (DAS) [38, 39] and the Wechsler Preschool and Primary Scale of Intelligence (WPPSI) [40]. The Vineland Adaptive Behavior Scale (VABS) [41, 42], a norm-referenced parent interview measure, was used in most of the studies to measure adaptive behavior. To assess autism severity, most studies used the Autism Diagnostic Observation Scale (ADOS) [43]. Other autism severity measures were the Childhood Autism Rating Scales (CARS) [44] and the parent-report Social Communication Questionnaire (SCQ) [45]. Measurement of early social and linguistic skills was the most variable, using language scales and a number of laboratory tasks and observational measures for gestures, joint attention, imitation, social reciprocity, and parent-child interaction (see Table 1).

#### 3.2.2. How Studies Controlled for Time 1 Ability Level Covariance with Age

Table 2 is organized around the four general research designs that were used across studies. It can be seen that this design aspect tended to determine the approach for investigating and controlling for the intercorrelations between age of starting early intervention and Time 1 ability levels. Studies that identify either younger- vs. older-starting groups at the beginning or higher and lower outcome groups at the end of intervention or after a follow-up period were more likely to directly examine early developmental levels associated with age.

#### 3.2.3. Results of Studies (Table 3)

Twelve of the 14 studies demonstrated that age of starting early intervention contributed significantly to outcome of children with ASD. The following describes the studies and their findings in this regard.

Studies that demonstrated no significant beneficial predictive effect of age of starting intervention or were equivocal are as follows:

1. Hedvall et al. (2015) [46]: this study examined children who had been treated with ABA-based early intervention in community programs. They identified a group who had gained the most (GM) (*N* = 30) and those who had lost the most (LM) (*N* = 23) over two years of intervention, based on a high and low 15^th^ percentile cutoff on outcome VABS scores. Those who had lost the most had been referred at an earlier age *and* examination of T1 attributes suggested that these children were more impaired initially. In a logistic regression of these predictors, only Time 1 cognitive level contributed independent variance to the outcome variable of adaptive behavior scores.
2. Tiura et al. (2017) [47]: this was a trajectory analysis study, and tracking patterns of change were associated with ABA intervention for 35 children aged 2–4 years at the beginning of the treatment. The measure used to track growth was the Developmental Profile-3^rd^ Edition (DP-3) [48], measuring its subdomains individually; these were administered every six months over three years. Multilevel modeling showed a linear growth model fit better than quadratic or cubic across the domains. In terms of predictors, the authors addressed the prediction/strength of association between all Time 1 variables first. However, these were not developmental quotients (which are ratios) but absolute age scores, and as a result, older children had higher scores. In their subsequent longitudinal prediction analyses, age of starting early intervention was not a significant predictor for outcome scores, whereas Time 1 ability levels such as cognitive and adaptive scores were. A second question was that of predicting growth rates. In this case, there was a nonsignificant trend for earlier age of entry to EI to predict faster growth rates.
3. Eapen et al. (2016) [49]: this study examined predictors of outcome in preschool children (*N* = 49) aged 3 to 5years when starting approximately 10 months of Early Start Denver Model (ESDM) group intervention. A linear regression controlled for baseline IQ, autism severity, and adaptive behavior. Initial age did account for a significant amount of the variance for autism severity as measured by parent-rated SCQ. On the other hand, this finding did not apply to outcomes that are usually considered important for demonstrating the effectiveness of intervention, that is, examiner-measured IQ, autism severity, and adaptive behavior. It is not evident why a parent-report measure is the only measure supporting the thesis.

Studies demonstrating the significant beneficial predictive effect of age of starting intervention using a variety of analytic strategies are as follows:

1. Kasari et al. (2012) [31]: the original study [50, 51] randomly assigned 58 preschoolers (aged 3 to 4 years) into two treatment groups and one nontreatment control group. For their later 2012 prediction study, reported on here, 40 of these children were re-examined five years later. The authors divided the children into those that reached a functional language level (approximately 2 years, 6-month level, *N* = 32) and those that did not (*N* = 8). A second grouping was those achieving a cognitive level at the same age equivalent (2 years, 6 months) versus not. Two other outcome targets were cognitive level and expressive vocabulary level (Expressive Vocabulary Test (EVT)) [52]. When a hierarchical multiple regression was then performed for the cognitive outcome, only baseline functional play level predicted the membership of the outcome group. However, *for level of spoken vocabulary at 8 to 9 years, starting age*, joint attention, play level, and treatment group assignment *all contributed significantly*. “*On average, the children gained a standard score of 1.1 (SE = 0.3) in spoken vocabulary ability per month that they entered the treatment earlier.*”
2. Smith et al. (2015) [32]: an intervention study of the Lovaas-based Young Autism Program was conducted as carried out in the community with a high level of supervision. Seventy-one children with ASD, aged 20 to 59 months, received two years of preschool-based intervention. The authors conducted sequential multiple regressions for each outcome measure separately for Year 1 and Year 2. The finding related to age of starting the intervention was as follows: controlling for all other covariates (IQ, adaptive skills, and autism severity), *lower age at intake predicted higher outcome for MSEL and, marginally, for VABS and ADOS scores*. An interaction effect was found for *Age* × *Year of Progress*, which is reported as follows.
3. Virues-Ortega et al. (2013) [53]: longitudinal multilevel modeling was used to identify predictors of outcomes of Intensive Behavioral Intervention (IBI) based on the Early Learning Accomplishment Profile (E-LAP) [54, 55] and the Learning Accomplishment Profile-Diagnostic, 3 Edition (LAP-D) [56]. Twenty-four children diagnosed with ASD (*M* = 50.0 months, *SD* = 28.3) participated in IBI over two years on average and were assessed using the ELAP every 6 months. A trajectory analysis demonstrated that the best solution for this relatively small sample was for two different groups, one higher performing over time and the other lower performing. The lower-performing children started with lower scores. In contrast, the higher-performing children, who started with higher scores, were less likely to decelerate and reach a ceiling in age-level measurement during the intervention period. Three child-associated variables were explored in two-predictor models. Age child started intervention best improved the fit for gross motor function, receptive language, self-care, and social behavior, while preintervention functioning level was the second most efficient predictor for regression models using fine motor function, prewriting, cognitive, and expressive language as outcome variables. Therefore, *regression analysis of predictors fitting to a growth curve showed that the best predictor was total intervention time, but the next best predictor was starting age, and after that, starting level of skills, depending on the outcome variable examined*.
4. Rogers et al. (2012) [57]: this study evaluated a 12-week, parent-delivered implementation of the Early Start Denver Model (ESDM) for 98 children aged 12 to 24 months. The children and families were randomized into either an ESDM treatment group or a TAU group. The intervention groups were combined to examine prediction relationships. The regression model held all other predictors constant as variables were added into the equation. *Age of starting intervention and number of intervention hours significantly predicted better outcomes at Time 2 for the sample as a whole, with IQ (MSEL) as the only outcome variable tested*.
5. Anderson et al. (2014) [58] reported on a long-term follow-up study that examined the factors at age 2 and 3 years that were associated with cognitive outcome at age 19, comparing those cognitively “more able” and “less able” (VIQ ≥ 70 or VIQ < 70 at Time 2) for 85 children diagnosed with ASD at age 2. To reduce the strong effects of Time 1 IQ when examining other types of predictive relationships, the researchers focused on two smaller groups: 32 youth with IQ ≥ 70, 24 with ASD, and eight who no longer had an ASD diagnosis (Very Positive Outcome or VPO). First, the two groups were compared on Time 1 scores to determine that they did not differ on baseline verbal IQ, nonverbal IQ, ADOS, and ADI-R scores, and thus, these features were unlikely to be confounds. Then, analyses conducted on early intervention experiences found that *those who no longer had ASD were more likely to have had EI between ages 2 and 3 compared to those who were in the ASD group*.
6. Vivanti et al. (2018) [59]: this was an RCT comparing ESDM in an inclusive classroom setting (*N* = 22) to one that was segregated, that is, only toddlers with ASD attended (*N* = 22). The follow-up period was nine months (one school calendar year). The role of age (when starting the program) was investigated for each group in predicting various outcome measures, including MSEL VDQ, NVDQ, video-rated language and social measures, an imitation task, and parent-rated ASD symptoms on the SCQ and the Repetitive Behavior Questionnaire-Revised (RBQ-R) [60]. The first set of analyses involved partial correlations between age of entry and each outcome variable, partialling out the Time 1 score for each variable. The one Time 2 variable that was significantly associated with starting age was MSEL Verbal DQ. The second approach was a linear regression. *Age of starting remained a significant predictor of VDQ after the variance associated with baseline VDQ and treatment group was accounted for*.

Studies demonstrating the significant beneficial predictive effect of age of starting intervention earlier versus later and generating an estimate of unique variance are as follows:

1. Perry et al. (2011) [27]: this study included primarily preschoolers (2 to 7 years old, *N* = 332), participating in a community-based, early IBI program with an average length of follow-up of 18 months. The researchers computed separate regressions for eight outcome variables (cognitive, adaptive, and autism severity measures) to see whether age contributed unique variance for each. At Step 1, they entered the Time 1 score for the same variable as a way of controlling for it and reported change in *R*^2^. Then, for each of the analyses, age was entered at Step 2, noting the increase in *R*^2^ and its significance. *Age of starting intervention accounted for a small, but significant, amount of unique variance for most of the outcome variables (from 1 to 6% of incremental variance)*.
2. A second approach used within this study was a hierarchical regression with a simultaneous solution using the variables of age, initial IQ, VABS ABC score, and CARS total score in predicting Time 2 IQ scores. Time 1 IQ was entered first and accounted for 54% of the variance. *Age at entry was then entered after baseline IQ and accounted for an additional 5.3% of the variance* (*p* < 0.001).
3. Kasari et al. (2012) [31]: this study was described above. In a separate hierarchical regression to predict spoken vocabulary at age 8, they input child age at entry to intervention (all spanned 3 to 4 years old), two early prelinguistic ability variables of initiating joint attention (IJA) and developmental play level and a treatment variable (treatment vs. no treatment). *Starting age predicted by itself 14% of the variance of later spoken vocabulary* (*p*=0.03). IJA, play level, and treatment assignment also each contributed unique variance (adding up to 50%).
4. Flanagan et al. (2012) [61]: this prediction study was carried out as a follow-up paper to previous intervention studies, including some subjects from the Perry et al. (2011) [27] study (see also [62]). Researchers reviewed the files of 61 age-matched pairs of children from a community-based EIBI program; one of each matched pair received IBI and the other was in a waitlist control group. Follow-up at Time 2 was after at least 12 months of intervention. Children in the IBI group were in treatment longer/were older at Time 2; otherwise, there were no differences between the two groups. The unique contribution of age was calculated in the context of a significant interaction. The regression analysis used one outcome variable—IQ at Time 2. The duration of treatment was entered at Step 1 because it differed between the two groups at baseline. Initial age was entered at Step 2 and was found to exert a significant effect, but with further steps, its unique contribution was diminished to nonsignificance. However, the interaction term of *Initial Age* × *Treatment Group*, at Step 7, was significant. In this context, *the amount of unique variance accounted for by age was 13%*.
5. Itzchak and Zachor, 2011 [63]: this study examined 78 children aged 15–35 months and evaluated predictors of outcome apart from treatment. Two groups received either early ABA or eclectic treatment. Predictors included child variables such as chronological age (CA) when starting intervention as well as the environmental variables of mother's age and education. Two hierarchical multiple regression analyses were performed using theoretically determined type and order of predictors. CA was included for the second regression analysis, with the change score in MSEL from Time 1 to Time 2 as the cognitive outcome. *When CA was entered at the third step, it accounted for 3% (p* < 0.05*) of the unique variance in the model.*

Studies demonstrating the role of age of starting early intervention through significant interaction effects are as follows.

In the following studies, the researchers were able to demonstrate, through the testing of interaction effects, more complex relationships among predictors when age was included in the interaction term.

1. Flanagan et al. (2012) [61]: this study was discussed above in the context of the percentage of unique variance of age in outcome prediction equations. The interaction term of *Initial Age* × *Treatment Group* was found to be significant, showing that younger age at initiation of treatment made a positive difference, but only if children were receiving IBI rather than TAU.
2. Vivanti et al. (2016) [64]: the differential impact of ESDM on the outcomes of younger children (18 to 48 months old) and older children (48 to 62 months old) was examined. Children received the ESDM for one year at 20 hours per week. An analysis of covariance (ANCOVA) was performed with Time 2 outcome as the within-subjects factor and age group (cutoff: above and below 48 months when starting intervention) as the between-subjects factor. Since the age groups did differ on Nonverbal developmental quotient (DQ), this was entered as the covariate. A significant *Age Group* *×* *Time* interaction was found, indicating that *while both groups showed a significant increase in Verbal DQ over the 12 months of treatment, children in the younger group experienced comparatively larger gains* (16 DQ points versus 7 points in the older group). This effect did not apply to Nonverbal DQ.
3. Smith et al. (2015) [32]: this study was discussed above as examining a community-based, two-year Young Autism Program with preschoolers. They entered an interaction term to evaluate the rate of progress in Year 1 and Year 2 in relation to age starting the program. A significant finding for the *Age* *×* *Time* interaction term showed that the progress slope was steeper for younger children, more so for Year 1 than Year 2. *Therefore, age made a difference in how children benefited from the intervention: children who were younger when starting made more progress in the first year of intervention than those who were older* (who also made progress, but not as much).

Studies showing that children made more gains in the beginning of the intervention (when they were younger) than in the later phases are as follows:

1. Smith et al. (2015) [32]: this study was discussed above as examining a community-based, two-year Young Autism Program with preschoolers, using regression analyses to evaluate the role of age as an individual predictor. They also entered an interaction term to examine the rate of progress in Year 1 and Year 2, and the nature of that progress for a younger vs. older group. They found that *all children made more initial progress in Year 1, with progress tapering off in Year 2, but that the younger a child was, the steeper the initial slope of progress.*
2. Virues-Ortega et al. (2013) [53]: this study used trajectory analysis and showed that the shape of the progress as shown in a learning curve was not linear for most children, but instead improved steeply in the beginning of their time in intervention and then plateaued. This applied more to children who started at a lower level of functioning than those who started with higher baseline scores. Analyzing the shape and course of the curves using “visual inspection,” it seemed evident that the higher-functioning children had steeper earlier curves and did not as frequently plateau during the intervention study time, but continued to make developmental gains. Regression analysis of predictors fitting to a growth curve showed that the best predictor was total intervention time, but the next best predictor was starting age, depending on the outcome variable. This suggests that *younger-starting children were more likely to have a steeper progress curve when intervention first began compared to older-starting children.*

### 3.3. Further Methodological Themes and Considerations

#### 3.3.1. Different Combinations of Predictors and Outcomes Gave Varied Results

Although there was general consistency among studies in terms of the constructs/variables examined, there were considerable differences in the pairing of predictors (apart from starting age of intervention) and outcomes. Even with similar sets of outcomes, significant results for the role of starting age were quite variable. For example, Virues-Ortega et al. [53] showed that the age children started intervention best improved (after the amount of time in intervention) the trajectory curve fit for gross motor function, receptive language, self-care, and social behavior, but preintervention level best improved the same regression model for outcome variables of fine motor function, prewriting, cognitive, and expressive language. Vivanti et al. [64] found a significant *Age Group* *×* *Time* interaction for Verbal DQ, but not for Nonverbal DQ. The majority of the studies showed a similar variety of results. The extent to which each specific domain was an outcome area affected by age of starting EI was calculated and is presented in Table 4. Averaging across all domains, earlier age made a positive difference about half the time.

#### 3.3.2. Specific Measurement Inconsistencies

One possible explanation for inconsistencies across studies when similar questions are asked may involve method and measurement choices. For cognitive measurement, there was a fair amount of consistency in instruments used, but then considerable inconsistency in which and how scores were employed for statistical analysis. For example, the MSEL was used in eight out of the fourteen studies. Yet five of the studies used ratio IQs made from the age equivalent scores in order to avoid the floor effect of the lowest standard score attainable being 20, while the two others used the standardized scale or composite scores from the manual tables. Across studies, there was a tendency to differentiate the MSEL verbal subscales from the nonverbal for both prediction and outcomes, yet some studies used the composite score. In community studies where there was less control over early assessment measures used, authors tended to equalize the cognitive scores by creating developmental quotients (DQs) based on age equivalents from various tests used. Most studies left out children for whom test scores could not be obtained.

Similarly, researchers made different decisions regarding specific VABS scores when addressing adaptive functioning. Some studies used age equivalents and others used standard scores; some used individual domains and others used the Adaptive Behavior Composite (ABC). There were other differences in terms of the edition used, and whether the researchers transformed the distribution or not.

Regarding autism severity, the three studies using the ADOS-T for either predictor or outcome variables each used the 1–10 severity score ratings, showing consistency in this way. In contrast, all three studies that focused on children under three years of age used the ADOS differently, ranging from the edition used to modifying the scores in an unconventional manner. Measurement of early other social and linguistic skills was highly variable, with little overlap among studies, making comparison difficult.

#### 3.3.3. Different Approaches to Data Analysis

In addition to the use of different predictors and outcomes that were measured through various instruments using a variety of scores, data analytic strategies were highly divergent. Each study using regression analysis employed a somewhat different approach for such procedures as choosing the candidate predictors, the number of predictors and outcome variables, the regression method per se, and reporting of *β* and ⊗ *R*^2^.

## 4. Discussion

Twelve out of the 14 studies reviewed did have at least one finding where earlier age of starting intervention was a statistically significant predictor of better developmental functioning and/or diagnostic status outcome in children with ASD. However, in the three studies where the unique variance for baseline age was reported, the amount was quite small (3% to 13%), especially in comparison with more robust predictors (usually T1 cognitive level). As well, the specific outcome measures that were predicted with statistical significance by initial age differed greatly among the studies.

More informative were findings that revealed more complex relationships among predictors when initial age was included. Three studies showed that initial age interacted with other variables, those of (1) effective treatment (younger age combined with more effective treatment (IBI) versus TAU made a positive difference in outcome); (2) more advanced initial language level (younger age combined with better early language was related to the better outcome); and (3) phase of intervention (children who were younger made more progress when they first started EI compared to older children).

This third finding—that the younger the children were, the more responsive they were to intervention, and that this response tapered off even at a young age—was evident throughout older to younger samples. Smith et al.'s (2015) [32] study included children as young as 20 months but primarily 3 to 5 years old, showing the effect for children who are not necessarily the youngest that participate in early intervention. The sample for Virues-Ortega et al. [53] was 25 months to 6 years, 5 months and produced similar effects. As the samples got younger, the effect continued to be found, for example, in four studies that included only children under 3 years of age.

Specifically, Anderson et al.'s (2014) [58] results indicated that receiving intervention between 24 and 36 months of age emerged as a difference between youth who no longer had an ASD diagnosis and those that retained an ASD diagnosis and had an average or above IQ. Orinstein et al. [65] had very similar findings, although their paper was not included in this review due to lack of control for baseline levels of several predictors. In these two studies, children generally did not start early intervention before age 24 months, but in the other two studies of the review that examined children only under age 3 years, some children were 14 to 15 months old when they started intervention. For example, children in the Itzchak and Zachor (2011) [63] study ranged from 15 to 35 months old, a span of 20 months, and baseline age was shown to account for a small amount of independent variance in the prediction of MSEL cognitive outcome. Rogers et al. [57], however, had a much younger group: 14–24 months, a spread of 10 months. Their results that age of starting intervention predicted MSEL IQ only three months later suggest that earlier is better could apply to the youngest children who are now receiving early intervention for ASD. The studies as a whole did not answer the question of when a relatively neuroplastic phase may no longer exert its influence, but they do suggest that the effect is operational in the youngest children.

Although this review intentionally focused on the variable of chronological age, attention to other predictors of outcome functioning was inevitable given that age interacted with or had relationships with these other variables. Up to this point in the literature, initial IQ or another early marker for overall ability level, possibly representing a neurological cap on developmental potential, usually has been considered to have the strongest influence on later functioning. This was the case for some of the studies in the current review [27, 31, 32]. However, in other studies that included predictors representing additional child characteristics on the one hand, and environmental variables such as type and intensity of treatment and demographics on the other hand, the overwhelming effect of initial IQ level was often mitigated. Nonetheless, the overriding effects of more severe delays could often be seen. From a methodological viewpoint, then, it will be important to take into account the number of children in a study sample with intellectual disability at Time 1, since the higher the proportion there is, the more difficult it will be to show differential prediction on other accounts.

The types of predictor variables examined in the reviewed studies were overwhelmingly child characteristics such as developmental or language level, adaptive behavior, social-communication skill levels, and autism severity, as well as child initial age. Yet child-focused/biologic variables and environmental risk and protective features are assumed to operate in a dynamic interplay, supporting or undermining development depending on timing, strength, and chronicity. The one environmental variable that was measured by all studies was treatment, since the studies were selected to include this feature. Several of the studies assessed parental variables such as educational and income level for the purposes of reporting sample characteristics and to demonstrate that groups being compared did not differ on these features. Only three of the studies included another environmental variable besides treatment as a predictor. Both Rogers et al. [57] and Eapen et al. [49] included a proximal parental variable reflecting treatment fidelity (e.g., how effectively the mother learned and used the interaction style that was taught during intervention) and it was found to influence the outcome positively. Itzchak and Zachor (2011) [63] found that the more distal variables of maternal age and education were significant predictors.

### 4.1. Other Key Methodological Issues

In 11 out of 14 of the studies, several different combinations of predictor and outcome variables were explored, with very inconsistent results across studies. What accounts for different positive versus negative findings given which predictors and which outcome variables were examined undoubtedly relates to both validity and reliability features of the measurement choices. A closer look at the specific scores used to represent the key constructs suggested that certain technical choices may be important for detecting reliable changes and true statistical relationships. For example, several authors have made detailed arguments for the use of age equivalents, especially in VABS adaptive behavior scores [32, 66].

For both VABS and MSEL, authors made different choices for examining subdomains rather than using composite scores.

Studies with young children with ASD increasingly differentiate verbal vs. nonverbal scores, with good reason, since language ability can be dissociated from the overall cognitive level across typical and disability populations. Studies also have shown the value of treating VABS domains (communication, daily living skills, etc.) as having different trajectories in autism populations presumably because the domains tap into different ability areas [66–68].

### 4.2. Implications for Treatment

The positive findings in the reviewed studies for “the earlier, the better” have implications for continued efforts for early identification and treatment. Even in the face of negative findings, there is a clear rationale for involving families with children with disabilities as early as possible in terms of support and advocacy skills that involvement with the intervention systems affords [69]. Most children with autism spectrum disorder will require first educational services and then vocational and adult support services. Therefore, the earlier caregivers learn what their children's needs are, what services they benefit from, and how the service systems work, the more able they will be to make decisions to support optimal development for their individual child [70].

Furthermore, the findings in these studies based on interactions among child characteristics have direct relevance to the current focus in the intervention literature regarding heterogeneous response to intervention. Translational questions have been posed regarding the difference between “rapid learners” vs. those with “poor response” [71], those who gained the most and lost the most [46], and responders vs. nonresponders [72]. Related to this is the question of which intervention approach is appropriate for which children. The themes emerging in the review regarding preintervention skill level, evidence-based intervention strategies, and amount of treatment, in addition to age at initiation of intervention, point to considerations for these important questions.

## 5. Conclusions and Future Directions

It is our assertion that this collection of studies and the current review constitutes a beginning evidence base for “earlier is better” with regard to interventions for young children with ASD.  Table 5 shows recommendations for future efforts that are examining whether earlier treatment initiation gives children with ASD an advantage in terms of later outcome. It is clear that this focus of inquiry will benefit from further research that is informed by these findings, as well as by the methodological strengths and limitations of the reviewed studies.

## Figures and Tables

**Table 1:** Methods features of studies reviewed.

Study	Research design	Sample size	Age (percent)			Follow-up	Variables and measurements				
			<3 yrs	3-4 yrs	>4 yrs	Period	Cognitive	Adaptive	ASD severity	Language	Social
*Studies with primarily preschoolers and older*											
Perry et al., 2011^1^	Intervention, pre-post	332	6.7	24	70	*M* = 18.4 months	DQs derived from different tests	VABS	CARS	N/A	N/A
Kasari et al., 2012^2^	Intervention, RCT 2 tx and 1 control group	40	15.9	68.2	15.9	5 years	DAS; SPA	N/A	ADOS	EVT	ESCS; PCX
Smith, Klorman, & Mruzek, 2015^3^	Intervention, pre-post	71	30.9	53.2	13.6	12 and 24 months	MSEL	VABS	ADOS; ADI-R	MCDI; ESCS	WSQ
Eapen, Crncec, & Walter, 2016	Intervention, pre-post	49	0.6	30.3	62.4	*M* = 9.8 months	MSEL	VABS	ADOS; SCQ	N/A	N/A

*Studies spanning age groups*											
Flanagan, Perry, & Freeman, 2012^1^	Intervention, waitlist/TAU control	142	30.9	38.2	28	*M* = 22.4 months	MSEL; WPPSI	VABS	CARS	N/A	N/A
Pellicano, 2012^4^	Longitudinal	37	40	37	23	Approx. 5 years *M* = 21.9 months	LIPS	N/A	ADI, ADOS-G, SCQ	PPVT-III	N/A
Virues-Ortega, Rodriguez, & Yu, 2013^3^	Intervention, pre-post and trajectory analysis	24	30.9	30	39.1	Approx. 2 years	E-LAP; LAP-D	E-LAP; LAP-D	N/A	E-LAP; LAP-D	E-LAP; LAP-D
Hedvall 2015^1^	Intervention, pre-post, two extreme groups	198	50	44	6	Approx. 2 years	DQs derived from different tests	VABS-II	BED, ABC	MCDI	VABS-II
Vivanti et al.,^3^ 2016	Intervention, pre-post	60	36.7	61.7	1.7	12 months	MSEL	VABS	ADOS-2	N/A	N/A

*Studies with infants and toddlers*											
Itzchak & Zachor 2011^3^	Intervention, two tx groups	78	100			12 months	MSEL	VABS	ADOS	MSEL VQ	N/A
Rogers et al., 2012^1^	Intervention, RCT	98	100			12 weeks	MSEL	VABS	ADOS-T	MCDI	Tasks
Anderson, Liang, & Lord, 2013^4^	Longitudinal	85	100			17 years	MSEL; DAS	VABS	ADOS; ADI-R	N/A	N/A
Tiura, Kim, Detmers, & Baldi, 2017^3^	Intervention, pre-post and trajectory analysis	35	78	12		3 to 6 years	DP-3	DP-3	High vs. low severity based on clinician's judgment	DP-3	DP-3
Vivanti et al., 2018^3^	Intervention, RCT, two tx groups	44	100			9 months	MSEL; imitation task	VABS	SCQ RBS = *R*	LENA	M-COSMIC

^1^Prediction study conducted after original intervention study was published. ^2^Prediction study conducted after additional follow-up period. ^3^Prediction analyses conducted concurrently with treatment effectiveness and presented in one publication. ^4^Longitudinal study; RCT = randomized controlled trial; TAU = treatment as usual; tx = treatment; DAS = Differential Ability Scales; SPA = Structured Play Assessment; MSEL = Mullen Scales of Early Learning; WPPSI = Wechsler Preschool and Primary Scale of Intelligence; LIPS = Leiter International Performance Scale; ADOS = Autism Diagnostic Observation Schedule; ADI-R = Autism Diagnostic Interview-Revised; VABS = Vineland Adaptive Behavior Scale; PPVT-III = Peabody Picture Vocabulary Test, 3^rd^ Edition; E-LAP = The Early Learning Accomplishment Profile; LAP-D = The Learning Accomplishment Profile-Diagnostic; BED = Best Estimate Diagnosis; ABC = Autism Behavior Checklist; CARS = Childhood Autism Rating Scale; SCQ = Social Communication Questionnaire; EVT = Expressive Vocabulary Test; MCDI = MacArthur–Bates Communicative Development Inventory; ESCS = Early Social Communication Scales; PCX = Parent-Child Interaction; WSQ = Wing Subtypes Questionnaire; DP-3 = Developmental Profile-3; RBS-R = Repetitive Behavior Scale-Revised; LENA = Language ENvironment Analysis; M-COSMIC = Modified Classroom Observation Schedule to Measure Intentional Communication.

**Table 2:** How studies addressed relationship of Time 1 ability levels and age of starting intervention.

Study	Did the study report on association between T1 cognitive, language, or adaptive scores and starting age?	How was the association of T1 cognitive, language, or adaptive scores and starting age controlled for?
*Studies explicitly comparing earlier- and later-starting children on outcomes after intervention*		
Vivanti, Dissanayake, & the ASEDCC team, 2016	The two groups of younger starting vs. older starting were compared on cognitive, verbal, and adaptive scores. Older children were found to have lower MSEL Nonverbal DQ scores	Thus, for the ANOVAs, Nonverbal DQ was entered as a covariate on all relevant analyses

*Studies comparing higher and lower outcome groups on T1 variables including starting age*		
Pellicano, 2012	The two groups of children with IQ > 80—one with ASD vs. no longer having ASD upon follow-up—were compared on cognitive and receptive language scores	Statistical analysis showed no T1 differences on PPVT-III and Leiter-R scores; in this way, these variables were held constant
Anderson, Liang, & Lord, 2014	The two groups of children with VIQ > 70—one with ASD vs. no longer having ASD (“very positive Outcome”)—were compared on adaptive scores at age 2 years	Statistical analysis showed no group differences at T1 in VABS scores; in this way, the variable was held constant
Hedvall et al., 2015	The two groups of children who had gained the most vs. lost the most from T1 to T2 were compared on adaptive and language scores at T1 as well as age at referral. Statistical analysis showed significant differences in VABS and MCDI scores, as well as age at referral	Logistic regression controlled for covariation of predictors

*Studies that added or focused on prediction during or after evaluation of an intervention*		
Itzchak & Zachor 2011	No	Regression analysis controlled for all other T1 variables including cognitive and adaptive scores
Perry et al., 2011	No	Regression analysis controlled for all other T1 variables including cognitive and adaptive scores
Flanagan, Perry, & Freeman, 2012	No	Sequential regressions entered intervention duration, age, and T1 adaptive skills, as well as other predictors and interaction terms, e.g., Age × Group
Kasari et al., 2012	No	First a forward regression procedure was used to identify strong predictors. Then hierarchical regression used predictors of age at the first assessment and play level, among others
Rogers et al., 20121	No	Linear regression analysis tested age of starting EI program controlling for all other predictors, including T1 cognitive, ADOS Social-Affect, and Imitation
Smith, Klorman, & Mruzek, 2015	No	Sequential multiple regression analysis entered T1 scores for the cognitive and adaptive outcome measure under analysis and age at intake. The interaction of Time × Age was also assessed, with effect of cognitive scores accounted for
Eapen, Crncec, & Walter, 2016	No	Linear regression analyses were conducted using predictors of T1 cognitive, adaptive, and play scores
Vivanti et al., 2018	No	First, a set of partial correlations were examined between age of entry and each outcome variable, partialling out the Time 1 score for each variable. Next, a linear regression entered age of starting the intervention after the variance associated with baseline Verbal DQ and treatment group was accounted for

*Trajectory analysis studies*		
Virues-Ortega, Rodriguez, & Yu, 2013	No	Multilevel regression analyses were used to predict an established growth curve, entering T1 scores to establish which accounted for the best fit and then which additional predictors added significantly to the goodness of fit. Predictors included pretreatment functioning level and age, among others
Tiura, Kim, Detmers, & Baldi, 2017	Yes, since they examined predictors for Time 1 cognitive levels to find that age of entry was significantly related. Children who were older when starting had higher cognitive levels	Multilevel regression analyses were used to predict an established growth curve, entering four T1 predictors to establish, first, which predicted Time 1 functioning levels, and second, which accounted for the best fit for the growth curves

**Table 3:** Results of studies and functioning areas showing beneficial effects of earlier starting age of intervention.

Study	Results showing significant predictive contribution of age of starting EI	Unique variance estimated for age of starting EI	Interaction effects that included starting age as predictor	Outcome variables affected by starting age				
				Cognitive (nonverbal)	Adaptive	ASD severity	Language	Social
*Studies with primarily preschoolers*								
Perry et al., 2011	Performed separate regressions for eight outcome variables: age contributed significantly for cognitive scores, autism severity, socialization, and motor skills when the baseline of the same variable at Time 1 was held constant. In further analyses of subgroups, such as those no longer having an ASD at outcome, results showed these participants, when first entering EI, tended to be younger and had higher IQs and less severe autism	N/A	N/A	Yes	N/A	N/A	N/A	N/A
Kasari et al., 2012	For the outcome of spoken vocabulary at age 8 years, child age at the beginning of the intervention, joint attention ability, play level, and treatment group assignment all contributed significantly. “On average, the children gained a standard score of 1.1 (SE = .3) in spoken vocabulary ability per month that they enter the treatment earlier”	Child age at the beginning of the study predicted by itself 14% of the variability of spoken vocabulary ability at age 8 (*p*=0.03)	N/A	No	N/A	N/A	Yes	N/A
Smith, Klorman, & Mruzek, 2015	Performed multiple regressions for each outcome measure, separately for Year 1 and Year 2. Controlling for other predictors, younger age at intake predicted higher outcome for MSEL and, marginally, for VABS and ADOS scores	N/A	Both younger and older children made more progress in Year 1 than in Year 2, but children who were younger when starting made even more progress in Year 1 than those who were older	Yes	No	No (Year 1)	N/A	No
				Yes	Yes	Yes (Year 2)	N/A	Yes
Eapen, Crncec, & Walter, 2016	Controlling for all other predictors (baseline IQ, autism severity, and adaptive behaviors) in a linear regression, initial age accounted for a significant amount of the variance for autism severity as measured by parent-rated SCQ, but not for other outcomes	N/A	N/A	No	No	Yes	No	Yes
*Studies spanning above and below three years of age*								
Flanagan, Perry, & Freeman, 2012	Performed regression analyses with one Time 2 outcome variable (IQ). Age × Treatment Group interaction term was significant (see column 3)	Used in an interaction term, age accounted for 13% of the variance	Being younger was an advantage only if children were in the Intensive Behavioral Intervention group rather than the treatment as usual group	Yes	N/A	N/A	N/A	N/A
Pellicano, 2012	At Time 2, all participants chosen for review had IQs > 80, but one group was determined to keep an ASD diagnosis and the other was deemed nonspectrum. These two groups did not differ on initial symptom level, language levels, or weekly hours of intervention. They did significantly differ on age of starting EI in that the nonspectrum group started earlier	N/A	N/A	N/A	N/A	Yes	N/A	N/A
Virues-Ortega, Rodriguez, & Yu, 2013	Regression analysis of predictors fitting to an established trajectory curve showed that total intervention time was the best predictor, but the second best was starting age, and after that, starting level of skills depending on outcome variable	N/A	N/A	No	Yes	N/A	Yes	Yes
Hedvall et al.,2015	From a larger group, 30 children who gained the most and 23 who lost the most over the intervention period were compared. Five predictors, including age at entry, when tested individually, showed significant relationships to outcome (adaptive behavior scores). However, results suggested younger children were more impaired. In a logistic regression of these predictors, only Time 1 cognitive level contributed independent variance	N/A	N/A	No	No	No	No	No
Vivanti et al., 2016	Intervention led to an increase in verbal scores for both younger and older age groups, but younger children made more gains. The regression analysis suggests that children who are older and have a lower language level (<13 months age equivalent) at entry made the least gains in Verbal DQ)	N/A	A significant Age Group × Time interaction showed that children in the younger group experienced comparatively larger gains after intervention (Nonverbal)	No (Nonverbal DQ)	No	No	Yes (Verbal DQ)	N/A

*Studies with infants and toddlers (all <3 years old)*								
Itzchak & Zachor, 2011	78 children were combined from an earlier study (ABA vs. eclectic intervention) to examine predictors of outcome apart from treatment. Predictors included child variables such as age as well as the environmental variables of mother's age and education. When age was included in one of the regression analyses, with the change score in MSEL from Time 1 to Time 2 as the cognitive outcome, it was a significant predictor	When age was entered at the third step, it accounted for 3% (*p*=0.05) of the unique variance in the model predicting cognitive change	N/A	Yes	No	N/A	N/A	N/A
Rogers et al.,2012	Regression procedure using Time 2 IQ as outcome showed age of starting intervention and number of intervention hours significantly predicted better outcomes for control and intervention groups combined	N/A	N/A	Yes (Nonverbal DQ)	N/A	N/A	N/A	N/A
Anderson, Liang, & Lord, 2014	Two groups (ASD with IQ > 70 vs. those who lost ASD diagnosis by age 19) were compared on Time 1 adaptive scores to determine that they did not differ. In this way, this baseline ability feature was held constant. Those who no longer had ASD were more likely to have had intervention between ages 2 and 3 years compared to those in the cognitively able ASD group	N/A	N/A	N/A	N/A	Yes	N/A	N/A
Tiura et al., 2016	Growth curve analysis was used to investigate predictive relationships to longitudinal changes of the ABA treatment outcomes. Relationships between Time 1 variables showed age of entry positively related to communication, cognitive, and adaptive levels. Age did not predict outcome levels of these variables. There was a nonsignificant trend for younger age to predict growth scores, however	N/A	N/A	N/A	No	N/A	No	No
Vivanti et al, 2018	The predictive role of starting age was examined for each of the two treatment groups (*N* = 22 each) for several distal and proximal child outcome variables. Partial correlations and linear regression showed that starting age was significantly and independently associated with NVQ such that the younger the starting age, at Time 1, the higher the NVQ at Time 2. This outcome was not achieved for any of the four other domains examined	N/A	N/A	No	No	No	Yes (Verbal DQ)	No

**Table 4:** Percentages of results across studies showing that earlier is better for domain outcomes.

	Outcome domain				
	Cognitive	Adaptive	ASD severity	Language	Social
# yes/# that tested	6/12	2/6	3/7	4/6	3/6
# no/# that tested	6/12	4/6	4/7	4/7	3/6
% earlier age predicted better outcomes (*M* = 49.4%)	54.5%	33.3%	42.8%	66.6%	50%

**Table 5:** Recommendations for research on the role of age of starting early intervention for children with autism spectrum disorder.

Area	Recommendations
Conceptual	(i) Planned analyses should be conceptually based
	(ii) Models will benefit from including both child-focused and environmental variables when considering the child variable of baseline age
	(iii) Conceptual models should take into consideration interaction of predictors

Participants	(i) Be aware of range of intellectual ability and proportion of severity levels present in the sample
	(ii) Include as broad a sampling as possible in terms of participant ability in cognition, play, language, and autism severity
	(iii) It would be helpful to have a more consistent definition of early intervention, distinguishing birth-to-three from preschool intervention
	(iv) School age (5 years and above) should not be considered early intervention

Measures	(i) When using standardized tests, consider value of age equivalent vs. standardized scores
	(ii) Explore ways to include children who cannot complete a standardized test
	(iii) Consider the constructs measured and separate out neuropsychological features such as language-based versus nonlanguage constructs and quotients

Approach to data analysis	(i) Explore distribution shape of continuous variables and adjust for skewness
	(ii) Testing of prediction relationships needs to move past zero-order correlations. Since starting age is an important theoretical predictor, lack of significant zero-order correlations may be bypassed for inclusion in further analysis because of the possibility of more complex relationships
	(iii) Multivariate approaches should control for shared variance among predictors
	(iv) Consider using statistical tests that are robust to small samples and nonparametric data (e.g., bootstrapping techniques) to minimize the possibility of type I and type II errors
	(v) Studies with large samples should consider more contemporary statistical approaches such as structural equation modeling in lieu of conducting multiple separate univariate and multivariate regression analyses
	(vi) Post hoc techniques for understanding the direction and magnitude of influence of age as predictor will be helpful
